# Spontaneous atopic dermatitis in mice with a defective skin barrier is independent of ILC2 and mediated by IL‐1β

**DOI:** 10.1111/all.13801

**Published:** 2019-04-29

**Authors:** Christian Schwartz, Tara Moran, Sean P. Saunders, Agnieszka Kaszlikowska, Achilleas Floudas, Joana Bom, Gabriel Nunez, Yoichiro Iwakura, Luke O’Neill, Alan D. Irvine, Andrew N. J. McKenzie, Graham Ogg, Patrick T. Walsh, Jocelyne Demengeot, Padraic G. Fallon

**Affiliations:** ^1^ School of Medicine Trinity Biomedical Sciences Institute, Trinity College Dublin Dublin Ireland; ^2^ Mikrobiologisches Institut ‐ Klinische Mikrobiologie, Immunologie und Hygiene Universitätsklinikum Erlangen and Friedrich‐Alexander Universität (FAU) Erlangen‐Nürnberg Erlangen Germany; ^3^ National Children’s Research Centre, Our Lady’s Children’s Hospital Dublin Ireland; ^4^ Instituto Gulbenkian de Ciência Oeiras Portugal; ^5^ Department of Pathology and Comprehensive Cancer Center University of Michigan Medical School Ann Arbor Michigan; ^6^ Research Institute for Biomedical Sciences Tokyo University of Science Chiba Japan; ^7^ School of Biochemistry and Immunology Trinity Biomedical Sciences Institute, Trinity College Dublin Dublin Ireland; ^8^ Department of Paediatric Dermatology Our Lady’s Children’s Hospital Dublin Ireland; ^9^ MRC Laboratory of Molecular Biology Cambridge UK; ^10^ MRC Human Immunology Unit, Weatherall Institute of Molecular Medicine John Radcliffe Hospital University of Oxford Oxford UK; ^11^ Trinity Translational Medicine Institute, St James’s Hospital, Trinity College Dublin Dublin Ireland

**Keywords:** atopic dermatitis, filaggrin, IL‐1β, innate lymphoid cells, microbiome

## Abstract

**Background:**

Atopic dermatitis (AD) is one of the most common skin diseases with a multifactorial etiology. Mutations leading to loss of skin barrier function are associated with the development of AD with group 2 innate lymphoid cells (ILC2) promoting acute skin inflammation. Filaggrin‐mutant (*Flg^ft/ft^*) mice develop spontaneous skin inflammation accompanied by an increase in skin ILC2 numbers, IL‐1β production, and other cytokines recapitulating human AD. Here, we investigated the role of ILC2, effector cytokines, inflammasome activation, and mast cell function on the development of chronic AD‐like inflammation in mice.

**Methods:**

Mice with a frameshift mutation in the filaggrin gene develop spontaneous dermatitis. *Flg^ft/ft^* mice were crossed to cell‐ or cytokine‐deficient mouse strains, or bred under germ‐free conditions. Skin inflammation was scored, and microbiome composition was analyzed. Skin protein expression was measured by multiplex immunoassay. Infiltrating cells were analyzed by flow cytometry.

**Results:**

Wild‐type and *Flg^ft/ft^* mice significantly differ in their microbiome composition. Furthermore, mutant mice do not develop skin inflammation under germ‐free conditions. ILC2 deficiency did not ameliorate chronic dermatitis in *Flg^ft/ft^* mice, which was also independent of IL‐4, IL‐5, IL‐9, IL‐13, IL‐17A, and IL‐22. Inflammation was independent of NLRP3 inflammasome activation but required IL‐1β and IL‐1R1‐signaling. Mechanistically, IL‐1β promoted hyperactivation of IL‐1R1‐expressing mast cells. Treatment with anti‐IL‐1β‐antibody alleviated dermatitis exacerbation, while antibiotic intervention ameliorated dermatitis in neonatal mice but not in adults with established inflammation.

**Conclusions:**

In summary, we identified a critical role for the microbiome and IL‐1β mediating chronic inflammation in mice with an impaired skin barrier.

## Graphical abstract

Filaggrin deficiency leads to skin dysbiosis early after birth altering adult immune responses, while mice raised under germ‐free conditions remain disease‐free. NLR Family Pyrin Domain Containing 3‐independent processing of IL‐1 in the skin promotes atopic dermatitis (AD)‐like ILC2‐independent inflammation. IL‐1 deficiency or targeting IL‐1 by monoclonal antibodies ameliorates dermatitis. IL‐1R1‐expressing dermal mast cells are key responders to IL‐1, acquire a hyperactive phenotype, and promote AD‐like inflammation.

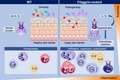

## 1 INTRODUCTION

Atopic dermatitis (AD) is a common eczematous pruritic disease with an onset at an early age, affecting up to 30% of children in the Western world. Etiology of AD is multifactorial including genetic predisposition, environmental factors, and immune status, leading to a high complexity in clinical presentation.^1^, ^2^, ^3^ Predisposing genetic factors for the development of AD include mutations in genes affecting the integrity of the skin barrier, such as mattrin (*TMEM79*) and filaggrin (filament aggregation protein, *FLG*).^4^, ^5^, ^6^ Filaggrin mutations were found in 30% of AD patients in Poland,^7^ China (26.0%),^8^ and Korea (15.7%),^9^ while healthy individuals had none. Importantly, filaggrin expression is downregulated in AD patients independent of their *FLG* genotype as a consequence of increased type 2 cytokines contributing to the aggravation of disease.^10^

We have previously separated and described the two mutated genes—Tmem79/mattrin and filaggrin—leading to the allergic skin phenotype of flaky tail mice.^5^, ^11^ Single mutant mice both have a defective skin barrier, and both spontaneously develop AD‐like inflammation. Pathogenesis in *Tmem79^ft/ft^* mice is dependent on adaptive immunity, while *Flg^ft/ft^* mice develop dermatitis through innate immune cells.^11^ However, the mechanisms underlying inflammation are unclear.

As an atopic disorder, AD is classically considered a type 2‐driven immunopathology involving type 2 T helper (Th2) cells, interleukin (IL)‐4, IL‐5, IL‐9, and IL‐13, as well as IgE, mast cells, basophils, and eosinophils—with more recent data expanding this view to include Th17 and IL‐22 cellular responses in the genesis of AD.^12^ While T cells are promoting inflammation in certain instances,^13^, ^14^, ^15^ they are largely dispensable in the *Flg^ft/ft^* model. Mast cells have long been associated with AD, and increased numbers are found in the skin of atopic patients.^16^ Upon activation by cytokines, FcεRI‐bound IgE, or pathogen‐ and danger‐associated molecular patterns, mast cells can release large amounts of pro‐inflammatory mediators, such as tumor necrosis factor (TNF).^17^, ^18^ Furthermore, increased numbers of group 2 innate lymphoid cells (ILC2) in the skin of *Flg^ft/ft^* mice and patients with mutations in *FLG* 11 suggest a central role for ILC2 in genesis of skin inflammation in this model. ILC2 are potent innate regulators of type 2 immune responses^19^, ^20^, ^21^, ^22^, ^23^, ^24^ and have been shown to promote inflammation in a model of acute dermatitis induced by topical application of MC903 (calcipotriol, vitamin D3 analogue).^25^, ^26^, ^27^, ^28^ However, their role in spontaneous dermatitis in *Flg^ft/ft^* mice is unknown.

Another hallmark of AD is skin dysbiosis, with a shift toward a pathogenic microbiome, in which beneficial commensals such as Propionibacteria or *Staphylococcus epidermidis* are displaced by other species such as *S. aureus*, and the patients’ overall skin microbiota diversity decreases.^29^, ^30^, ^31^, ^32^ Results from flaky tail mice (*Flg^ft/ft^Tmem79^ft/ft^*) suggested that the microbiota promotes upregulation of IL‐17A and the infiltration of neutrophils and eosinophils into the skin.^33^

Polymorphisms in members of the IL‐1 family of cytokines and their receptors are associated with skin disorders, such as cutaneous lupus erythematosus, psoriasis, and atopic dermatitis.^34^, ^35^ We have previously reported increased IL‐1α, IL‐1β, and IL‐1R1 expression in skin of *Flg^ft/ft^* mice and AD patients with mutations in *FLG*.^36^

In the present study, we set out to investigate the mechanisms underlying AD‐like inflammation in *Flg^ft/ft^* mice. We discovered that ILC2—while required for acute MC903‐induced dermatitis—were dispensable for spontaneous AD‐like inflammation in *Flg^ft/ft^* mice with an impaired skin barrier. Instead, the development of skin inflammation was dependent on an interplay between microbiota, IL‐1β, and mast cells.

## 2 MATERIAL AND METHODS

### 2.1 Mice

The following mice were backcrossed onto the *Flg^ft/ft^* (initially isolated from flaky tail mice, JR#9078, Jackson Laboratories, Bar Harbor, ME^11^) BALB/c background for >8 generations: *Rag1^−/−^* (JAX: 002216),^37^ *Rag2^−/−^γc^−/−^* 38 (JAX; 014593), *Il7r^Cre^*,^39^ Rora^flox^,^23^ sg/sg mice,^40^ *Il4^KN2^*,^41^ *Il5^cer/cer^*,^11^ *ll13^−/−^*,^42^ *Il9^cit/cit^*,^43^ *Il17a^−/−^*,^44^ *Il22^−/−^*,^45^ *Nlrp3^−/−^*,^46^ *Il1a^−/−^*,^47^ *Il1b^−/−^* ^47^, *Il1r1^−/−^* (JAX:003245),^48^ *Il18^−/−^* (JAX:004130),^49^ *Asc^−/−^*,^50^ *Aim2^−/−^*,^51^ *Kit^W‐sh/W‐sh^* (JAX:005051),^52^ and *Il36r^−/−^*.^53^ Mice were housed in a specific pathogen‐free facility, with irradiated diet and water ad libitum. Experiments under germ‐free conditions were conducted at the Instituto Gulbenkian De Ciência in Portugal. Animal experiments were approved by Trinity College Dublin BioResources and Instituto Gulbenkian de Ciência ethical review board and performed in compliance with EU Directive 2010/63/EU, Irish Medicine's Board and The Health Products Regulatory Authority.

### 2.2 Scoring of skin inflammation

Severity of skin inflammation was clinically scored (total range: 0‐12) by macroscopic diagnostic criteria as previously described.^5^ The total score is the sum of individual scores ranging from 0 to 3 (0, none; 1, mild; 2, moderate; 3, severe) that were applied to edema, erythema, scaling, and erosion.

### 2.3 Preparation of bone marrow–derived mast cells (BMMC) and stimulation

Bone marrow was isolated from the femur and tibia of donor mice and cultured in media (RPMI + 10% FBS + L‐glutamine + penicillin/streptomycin + HEPES + non‐essential amino acids) containing 10 ng/mL SCF and 10 ng/mL IL‐3 (R&D systems) for a total of 4 weeks with media changes twice a week.^54^, ^55^ 3 × 10^5^ mast cells/well were left untreated or were stimulated with plate‐bound anti‐FcεRIα (10 µg/mL, clone MAR‐1, Thermo Scientific) in the presence or absence of IL‐1β (10 ng/mL, R&D systems) for 24 hours. For adoptive transfers, BMMC were resuspended in sterile PBS. Mice received 1 × 10^6^ mast cells via intradermal injection into the ear.

### 2.4 Microbiome analysis

Skin microbiome samples were acquired by exposing sterile swabs to the ear skin of *Flg^ft/ft^* and wild‐type mice, as previously published.^56^ Mice were kept in the same or adjacent cages, looked after by the same person using the same products. Same surface area was sampled for all age‐ and sex‐matched mice. To avoid cross‐contamination, sterile gloves were changed between each sample collected. Samples were instantly frozen in liquid nitrogen, and 16S rRNA gene sequencing and microbiome analysis was performed by Second Genome (San Francisco, CA), as previously described.^57^

### 2.5 Axenic mouse model generation

Male and female *Flg^ft/ft^* mice were shipped from Trinity College Dublin to the Instituto Gulbenkian De Ciência in Portugal and re‐derived by embryo transfer from a quarantine facility into SPF housing. Subsequent litters were generated by timed‐pregnancies. Fetuses were transferred to GF isolators and fostered by GF C3H mothers, as described in the relevant EMMA protocol (http://strains.emmane-t.org/protocols/GermFree_0902.pdf). Germ‐free and age‐matched SPF control litters were raised and maintained under strictly identical conditions (food, water, humidity, temperature), except the microbiological status.

### 2.6 Statistics

GraphPad Prism (version 7) was used to generate graphs and for statistical analysis. Area under curve (AUC), Student's *t* test, and ANOVA were used to determine statistical significance. *P*‐values < 0.05 were considered statistically significant.

Please refer to the Supporting Information for additional materials and methods.

## 3 RESULTS

### 3.1 Flg^ft/ft^ mice develop spontaneous atopic dermatitis–like skin inflammation

**Figure 1:**
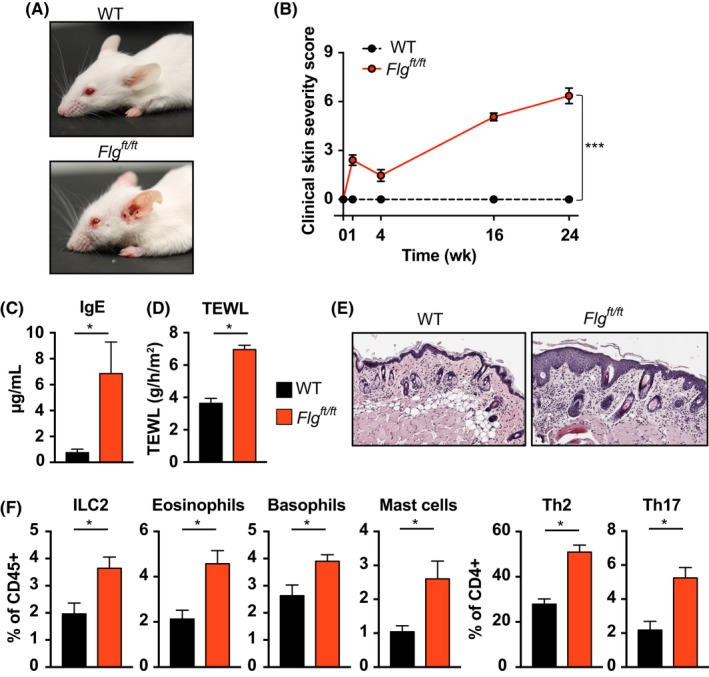
*Flg^ft/ft^* mice develop spontaneous atopic dermatitis associated with increased cellular infiltration. A, Representative photographs of wild‐type Balb/c (WT, top) and filaggrin‐mutant *Flg^ft/ft^* mice (bottom) at ten weeks of age. B, Macroscopic clinical scoring of Balb/c (black) and *Flg^ft/ft^* (red). Graph shows the mean ± SEM from 20 mice per group. ***, *P* < 0.001, *t* test of AUC. C, Total serum IgE concentrations from adult wild‐type (black) and 12‐wk‐old *Flg^ft/ft^* mice (red). Bars show the mean ± SEM of seven mice. *, *P* < 0.05. D, Transepidermal water loss (TEWL) at 12 wk in wild‐type (black) and *Flg^ft/ft^* mice (red). Bars show the mean ± SEM of nine mice. *, *P* < 0.05. E, Representative photomicrograph of H&E‐stained skin from *Flg^ft/ft^* (right) and wild‐type mice (left). 20x original magnification. (F) Frequencies of indicated cell types isolated from ear skin of wild‐type (black bars) and *Flg^ft/ft^* mice (red bars) and analyzed by flow cytometry. Bars show the mean + SEM of 6‐8 mice per group of two independent experiments. *, *P* < 0.05

We sought to investigate the innate mechanisms that elicit inflammation in *Flg^ft/ft^* mice. *Flg^ft/ft^* mice develop spontaneous skin inflammation as neonates, with a second phase of overt inflammation—most prominent around the eyes and ears—progressing from 8 weeks of age evidenced by a constant increase in clinical severity (Figure 1A,B). The impaired skin barrier in *Flg^ft/ft^* mice is represented by significantly increased transepidermal water loss (TEWL), and development of skin inflammation is accompanied by increased circulating IgE (Figure 1C,D). Skin histology of *Flg^ft/ft^* mice shows dermal and epidermal thickening, scaling, and cellular infiltration into the skin (Figure 1E). Among skin infiltrating immune cells, we find significantly increased numbers of ILC2, eosinophils, basophils, Th2 cells, and also mast cells (Figure 1F)—similar to the localized immune cell repertoire in AD patients.^11^, ^27^, ^58^, ^59^, ^60^

### 3.2 Pathogenic cutaneous microbiome promotes chronic inflammation in Flg^ft/ft^ mice

**Figure 2:**
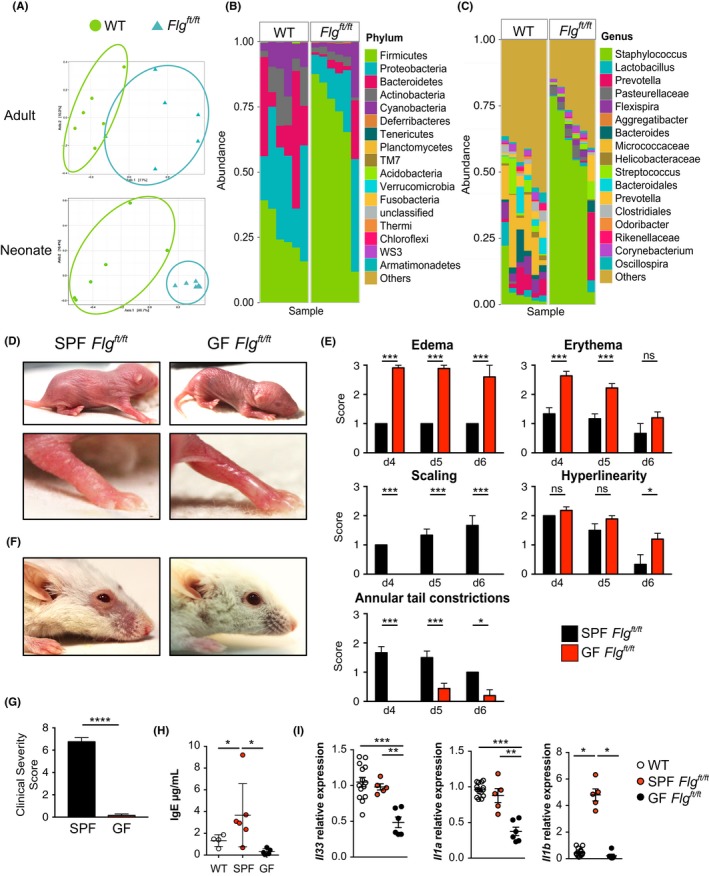
Pathogenic cutaneous microbiome promotes chronic inflammation in *Flg^ft/ft^* mice. A, Principal component analysis of microbiome samples isolated from skin swaps of wild‐type (blue triangles) and *Flg^ft/ft^* mice (green circles). Non‐lesional ear skin was swabbed at 12 wk (upper panel) and 3‐4 d (lower panel) of age. B,C, Mean relative abundance of bacterial phyla (B) and genera (C) of bacteria colonizing the skin of adult wild‐type and *Flg^ft/ft^* mice. D, Representative photographs of neonatal *Flg^ft/ft^* mice in a specific pathogen‐free (SPF, left panels) or germ‐free (GF, right panels) environment. E, Macroscopic scores for edema, erythema, scaling, hyperlinearity, and annular tail constrictions in neonatal SPF (black) and GF (red) *Flg^ft/ft^* mice. Bar graphs show the mean + SEM of 9 mice per group. ns, not significant; *P < 0.05, ***P < 0.001. F, Photograph of adult *Flg^ft/ft^* raised in an SPF (left) or GF (right) environment. Representative of 9 mice per group. G, Macroscopic scoring of SPF (black) and GF (red) *Flg^ft/ft^* adult mice. ***P < 0.001. H, Serum IgE concentrations in SPF WT (open circles), SPF *Flg^ft/ft^* (red), and GF *Flg^ft/ft^* (black) adult mice. I, Relative quantification of *Il33, Il1a*, *Il1b* in the skin of adult WT (open circles) and adult *Flg^ft/ft^* mice raised under SPF (red) or GF (black) conditions. Bars show the mean + SEM of 6 mice per group

In order to investigate whether *Flg^ft/ft^* mice faithfully reflect the dysbiosis apparent in the skin microbiome of AD patients, we analyzed their skin microbiota. 16S rRNA‐sequencing revealed a significantly altered microbiome in both adult and neonatal *Flg^ft/ft^* mice compared to wild‐type animals kept in the same facility (Figure 2A). The defective skin barrier integrity in *Flg^ft/ft^* mice led to decreased microbial diversity with pronounced overrepresentation of firmicutes (Figure 2B), including *Staphylococcus* species (Figure 2C). Strikingly, *Flg^ft/ft^* mice raised in germ‐free (GF) conditions developed marked skin inflammation as neonates (Figure 2D,E) that resolved in adults (Figure 2F,G). Consistent with the lack of evident pathology in adult GF mice, serum IgE was reduced to WT level (Figure 2H). Expression of genes associated with AD (*Il33, Il1a, Il1b*) was significantly altered in the absence of microbiota (Figure 2I). Importantly, while *Il33* and *Il1a* were downregulated independent of filaggrin deficiency, *Il1b* was significantly increased in the skin of 12‐week‐old adult *Flg^ft/ft^* mice. *Il1b* expression was restored to WT levels when mice were kept under germ‐free conditions (Figure 2I). Similarly, treatment of pregnant *Flg^ft/ft^* females and their litters with broad‐spectrum antibiotics (ABX) from day E14 to P21 significantly decreased clinical severity of skin inflammation in adult offspring at 12 weeks of age (Figure S1A). In contrast, when adult 8‐9‐week‐old *Flg^ft/ft^* mice that had already developed skin inflammation were treated with ABX for 4 weeks, there was no amelioration of dermatitis (Figure S1B). These data indicate that the development of AD‐like skin inflammation in *Flg^ft/ft^* mice is microbe‐mediated in neonatal stages, while in adult animals—once skin inflammation is established—antibiotic intervention cannot alter skin disease. However, the cellular events leading up to AD development in this mouse model remain unclear.

### 3.3 Atopic dermatitis is independent of group 2 innate lymphoid cells

**Figure 3:**
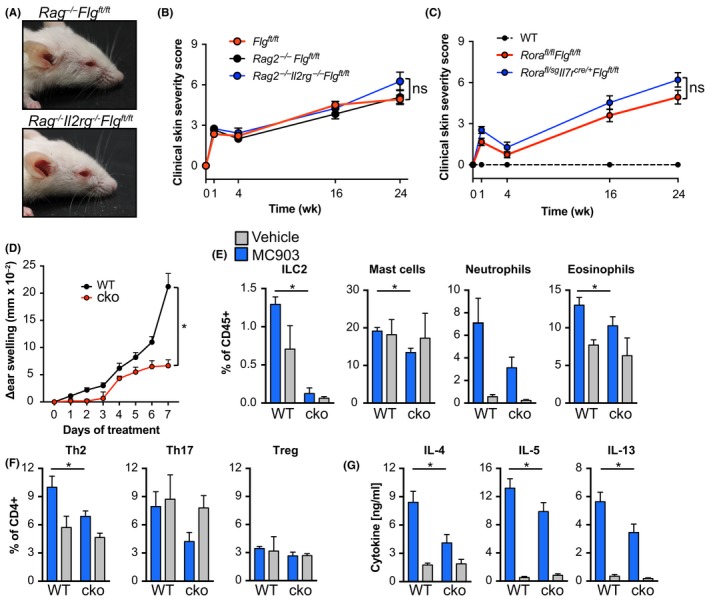
Dermatitis in *Flg^ft/ft^* mice is independent of ILC2, while acute skin inflammation is ILC2‐dependent. A, Representative photographs of *Rag^−/−^Flg^ft/ft^* (upper panel) and *Rag2^−/−^Il2rg^−/−^Flg^ft/ft^* (lower panel) mice. B, Macroscopic clinical scoring of *Flg^ft/ft^* (red), *Rag2^−/−^Flg^ft/ft^* (black), and *Rag2^−/−^Il2rg^−/−^Flg^ft/ft^* (blue). Graph shows the mean ± SEM from 6 to 7 mice per group. C, Macroscopic clinical scoring of wild‐type (dashed black), *Rora^fl/fl^Flg^ft/ft^* (red), and *Rora^fl/sg^Il7r^Cre/+^Flg^ft/ft^* (blue) mice. Graph shows the mean + SEM from 9 mice per group. D, Skin inflammation was induced in *Rora^fl/sg^Il7r^Cre/+^* (cko; red line) and control (WT; black line) mice by daily topical application of 4 nmol MC903 in 100% ethanol onto the right ear. The left ear was treated with ethanol and served as internal control. Ear thickness was measured daily. Mean ± SEM from at least 6 mice per group from two independent experiments is depicted. *P < 0.05, *t* test of AUC. E, Frequency of ILC2, mast cells, neutrophils, and eosinophils isolated from the ears of MC903‐ (blue) and ethanol‐treated (Vehicle, gray) *Rora^fl/sg^Il7r^Cre/+^* (cko) and control (WT) mice. F, Frequency of T helper cell subsets isolated from the ears of MC903‐ (blue) and ethanol‐treated (gray) *Rora^fl/sg^Il7r^Cre/+^* (cko) and control (WT) mice. Bar graphs show the mean ± SEM from six mice of two independent experiments. *P < 0.05. G, Draining (MC903, blue) and non‐draining (Vehicle, gray) lymph node cells were restimulated with anti‐CD3/anti‐CD28 for 72 h and indicated cytokines in the supernatant were measured by ELISA. Bar graphs show the mean + SEM from 6 to 8 mice per group from two independent experiments. ns, not significant, *t* test of AUC

The appearance and severity of dermatitis in *Flg^ft/ft^* mice is dependent on cells of the innate immune system (Figure 3A)^11^ with a significant increase in dermal ILC2 numbers in *Flg^ft/ft^* mice (Figure 1F). Therefore, we tested whether ILC‐deficient mice on *Flg^ft/ft^* background developed AD. Treatment of *Rag1^−/−^Flg^ft/ft^* mice with anti‐CD90 mAb to deplete ILC2 did not alter development of skin inflammation (data not shown). *Rag2^−/−^Il2rg^−/−^Flg^ft/ft^* mice developed skin inflammation comparable to *Flg^ft/ft^* mice and *Rag2^−/−^Flg^ft/ft^* mice (Figure 3B). As *Rag2^−/−^Il2rg^−/−^* are not only deficient in ILC but also NK, T, and B cells, we wanted to confirm our results by using a more specific model of ILC deficiency. Therefore, ILC2‐deficient *Rora^fl/sg^Il7ra^Cre/+^* mice^23^ were crossed onto the *Flg^ft/ft^* background. ILC2‐deficient *Flg^ft/ft^* mice developed severe skin inflammation (Figure 3C). These data from three separate and distinct models of ILC2 deficiency led us to conclude that ILC2 were dispensable for skin inflammation in this spontaneous and chronic model of AD‐like inflammation in mice with a defective skin barrier.

### 3.4 Group 2 innate lymphoid cells promote acute skin inflammation

Daily application of MC903 elicits acute AD‐like skin inflammation^28^ (Figure 3D). We, and others, have previously shown using antibody‐mediated depletion models as well as bone marrow chimeric mice that development of MC903‐elicited skin inflammation is dependent on ILC2.^25^, ^27^ Indeed, using ILC2‐deficient *Rora^fl/sg^Il7ra^Cre/+^* mice, we could confirm that ear swelling was ameliorated in acute dermatitis (Figure 3D). As expected, ILC2 were not present in the inflamed skin of *Rora^fl/sg^Il7ra^Cre/+^* mice (Figure 3E). Moreover, cellular infiltration was blunted in ILC2‐deficient mice including the recruitment of granulocytes, Th2 and Th17 cells (Figure 3E,F). Furthermore, type 2‐associated cytokine production in draining LN was impaired (Figure 3G). Because of the divergent roles ILC2 play in the initiation processes of acute chronic dermatitis, we sought to determine which other factors promote AD in the clinically more relevant *Flg^ft/ft^* model.

### 3.5 Impaired skin barrier–induced dermatitis operates independently of type 2 and type 17 cytokines

**Figure 4:**
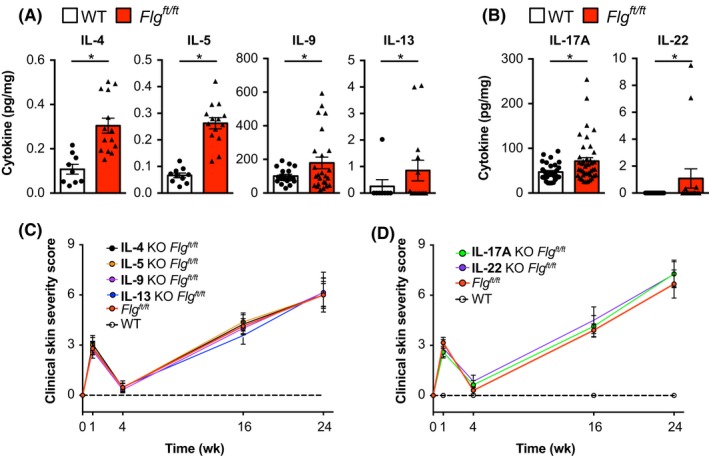
Dermatitis in *Flg^ft/ft^* mice is independent of type 2‐ and type 17‐associated effector cytokines. A,B, Indicated cytokines isolated from ear skin of age‐matched wild type (open bars) and *Flg^ft/ft^* (red bars) depicted as pg cytokine per mg of total isolated protein. C, Macroscopic clinical scoring of wild‐type (dashed black), *Flg^ft/ft^* (red), IL‐4*^−/−^Flg^ft/ft^* (black), IL‐5*^−/−^Flg^ft/ft^* (orange), IL‐9*^−/−^Flg^ft/ft^* (violet), and IL‐13*^−/−^Flg^ft/ft^* (blue) mice. Graph shows the mean + SEM from at least 8 mice per group. D, Macroscopic clinical scoring of wild‐type (dashed black), *Flg^ft/ft^* (red), IL‐17A*^−/−^Flg^ft/ft^* (green), and IL‐22*^−/−^Flg^ft/ft^* (violet) mice. Graph shows the mean + SEM from at least 6 mice per group from two to three independent experiments

Atopic dermatitis in humans is generally accompanied by an increase in type 2‐ and type 17‐associated cytokines,^61^, ^62^, ^63^ with IL‐13 and IL‐5 reported as important systemic biomarkers for infant AD.^64^ Indeed, analysis of cytokines in the skin of *Flg^ft/ft^* mice revealed a significant increase of IL‐4, IL‐5, IL‐9, IL‐13, IL‐17A, and IL‐22 (Figure 4A,B). To investigate whether the development of AD‐like inflammation in *Flg^ft/ft^* mice was dependent on the cardinal type 2 cytokines, we generated IL‐4‐, IL‐5‐, IL‐9‐, and IL‐13‐knockout mice on the *Flg^ft/ft^* background. However, Th2 cytokine‐deficient *Flg^ft/ft^* mice developed inflammation comparable to that observed in cytokine‐sufficient *Flg^ft/ft^* mice (Figure 4C). Similarly, knockout of IL‐17A or IL‐22 on the *Flg^ft/ft^* background did not ameliorate skin inflammation (Figure 4D). Because neither knockout of individual type 2 nor type 17 cytokines protected from skin inflammation, we investigated cytokines upstream in the inflammatory cascade.

### 3.6 Inflammasome‐independent IL‐1β‐mediated IL‐1R1 signaling is required for inflammation

**Figure 5:**
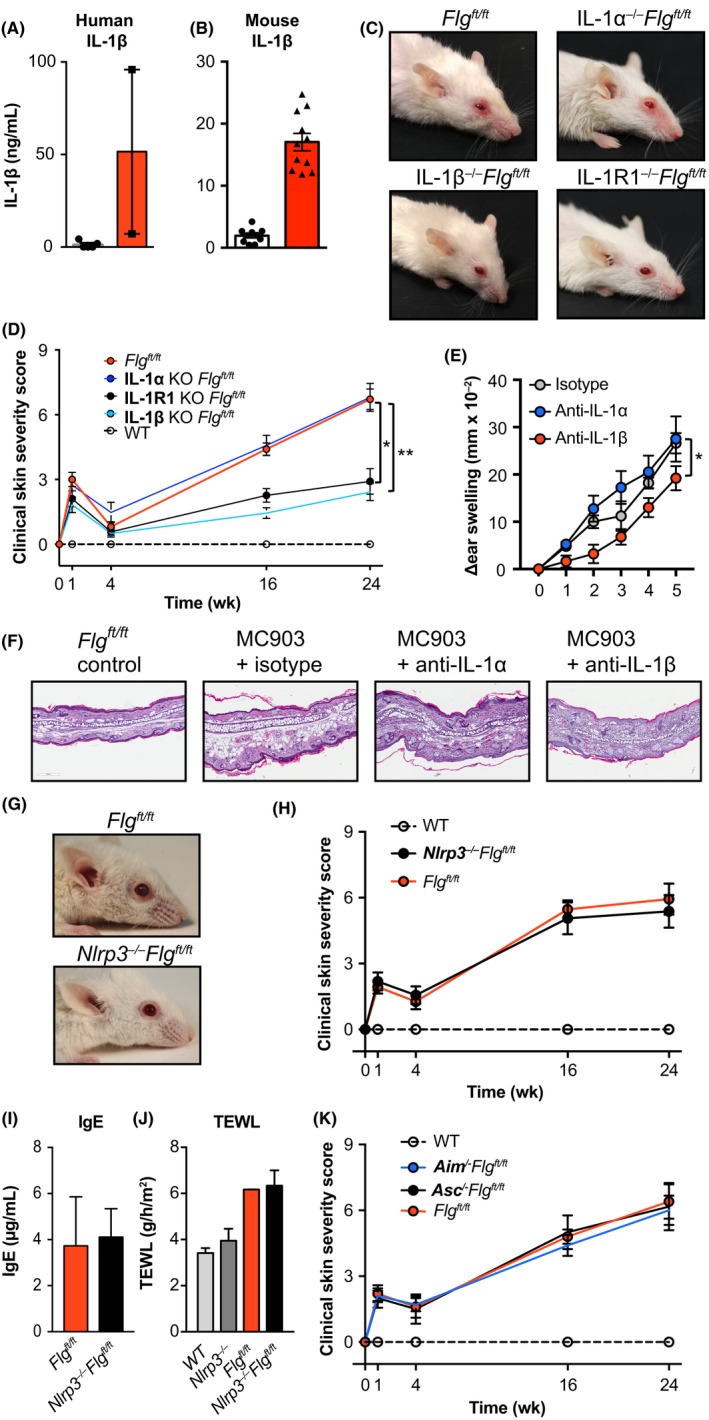
Inflammation in *Flg^ft/ft^* mice is dependent on IL‐1β and IL‐1R1 signaling but occurs inflammasome‐independent. A, IL‐1β was measured in skin biopsies of patients with or without FLG mutation. B, IL‐1β protein concentration in ear skin of age‐matched wild type (open bars) and *Flg^ft/ft^* (red bars) depicted as pg cytokine per mg of total isolated protein. C, Representative photographs of *Flg^ft/ft^* (upper left), IL‐1α*^−/−^Flg^ft/ft^* (upper right), IL‐1β*^−/−^Flg^ft/ft^* (lower left), and IL‐1R1*^−/−^Flg^ft/ft^* (lower right). D, Macroscopic clinical scoring of wild‐type (open circles), *Flg^ft/ft^* (red), IL‐1R1*^−/−^Flg^ft/ft^* (black), IL‐1β*^−/−^Flg^ft/ft^* (light blue), and IL‐1α*^−/−^Flg^ft/ft^* (blue) mice. Graph shows the mean + SEM from 8 mice per group. *P < 0.05; **P < 0.01; *t* test of AUC against *Flg^ft/ft^*. E, *Flg^ft/ft^* mice were treated daily with isotype control mAb (gray circles), anti‐IL‐1α‐(blue) or anti‐IL‐1β‐mAb (red) and MC903 was topically applied to induce dermatitis exacerbation. Ear thickness was measured daily. Graph shows the mean + SD of 4‐5 mice per group. *P < 0.05, AUC. F, Representative H&E‐stained sections of untreated (left) and MC903‐treated ears from (E). G, Representative photographs of *Flg^ft/ft^* (upper panel) and *Nlrp3^−/−^Flg^ft/ft^* (lower panel) mice. H, Macroscopic clinical scoring of wild‐type (open circles), *Flg^ft/ft^* (red), and *Nlrp3^−/−^Flg^ft/ft^* (black) mice. Graph shows the mean + SEM from 7 mice per group. I, Serum IgE levels in *Flg^ft/ft^* (red), and *Nlrp3^−/−^Flg^ft/ft^* (black) mice. J, Transepidermal water loss in wild‐type (gray), *Nlrp3^−/−^* (dark gray), *Flg^ft/ft^* (red), and *Nlrp3^−/−^Flg^ft/ft^* (black) mice. Bar graphs show the mean + SEM from 4 to 6 mice per group. K, Macroscopic clinical scoring of wild‐type (open circles), *Flg^ft/ft^* (red), *Aim^−/−^Flg^ft/ft^* (blue), and *Asc^−/−^Flg^ft/ft^* (black) mice. Graph shows the mean + SEM from 6 to 10 mice per group from three independent experiments

IL‐1β is significantly elevated in skin blisters of patients with AD that have *FLG* mutations (Figure 5A) and in the skin of *Flg^ft/ft^* mice (Figure 5B). We crossed *Il1a^−/−^*, *Il1b^−/−^,* and *Il1r1^−/−^* mice onto the *Flg^ft/ft^* background to investigate their contribution to spontaneous skin inflammation (Figure 5C,D). While IL‐1α deficiency did not decrease the inflammatory score of *Flg^ft/ft^* mice (*P* = 0.942), IL‐1β‐deficient *Flg^ft/ft^* mice were protected from the development of skin inflammation (Figure 5C,D; *P* = 0.013). Importantly, IL‐1R1 was required for IL‐1β‐mediated inflammation as *Flg^ft/ft^* mice deficient in IL‐1R1 were comparably protected (Figure 5C,D; *P* = 0.009). Furthermore, inhibition of IL‐1β by treatment with anti‐IL‐1β‐antibodies ameliorated MC903 elicited acute exacerbation of skin disease in *Flg^ft/ft^* mice (Figure 5E,F). We addressed contribution of other IL‐1 cytokine family members to spontaneous skin inflammation in *Flg^ft/ft^* mice but development of dermatitis in *Flg^ft/ft^* mice was independent of IL‐33, IL‐18, and IL‐36 (Figure S2A). These results reveal an important role for IL‐1β and IL‐1R1 in the development of spontaneous skin inflammation in this model.

IL‐1β requires processing by the NLRP3 inflammasome to be cleaved from pro‐IL‐1β to become bioactive IL‐1β. We generated *Nlrp3^−/−^Flg^ft/ft^* mice to investigate the contribution of the NLRP3 inflammasome to inflammation in our model (Figure 5G,H). However, inflammasome‐mediated maturation was not required as these mice developed skin inflammation comparable to that seen in *Flg^ft/ft^* mice (Figure 5G,H). NLRP3 deficiency did not alter the defective skin barrier or generation of IgE in mutant mice (Figure 5I,J). Indeed, there was also no role for ASC and AIM2 (Figure 5K). We further addressed whether inflammation in *Flg^ft/ft^* mice can be targeted therapeutically with the potent inflammasome inhibitor MCC950^65^ during a period of MC903‐induced exacerbation of skin inflammation. Chemical inhibition of the inflammasome did not ameliorate the acute development of skin inflammation in *Flg^ft/ft^* mice (Figure S2B), which was consistent with our results on spontaneous and chronic inflammation in *Nlrp3^−/−^Flg^ft/ft^* mice. These data demonstrate the development of skin inflammation in mutant mice is independent of the NLRP3 inflammasome.

### 3.7 Dermal mast cells promote inflammation in mice with impaired skin barrier

**Figure 6:**
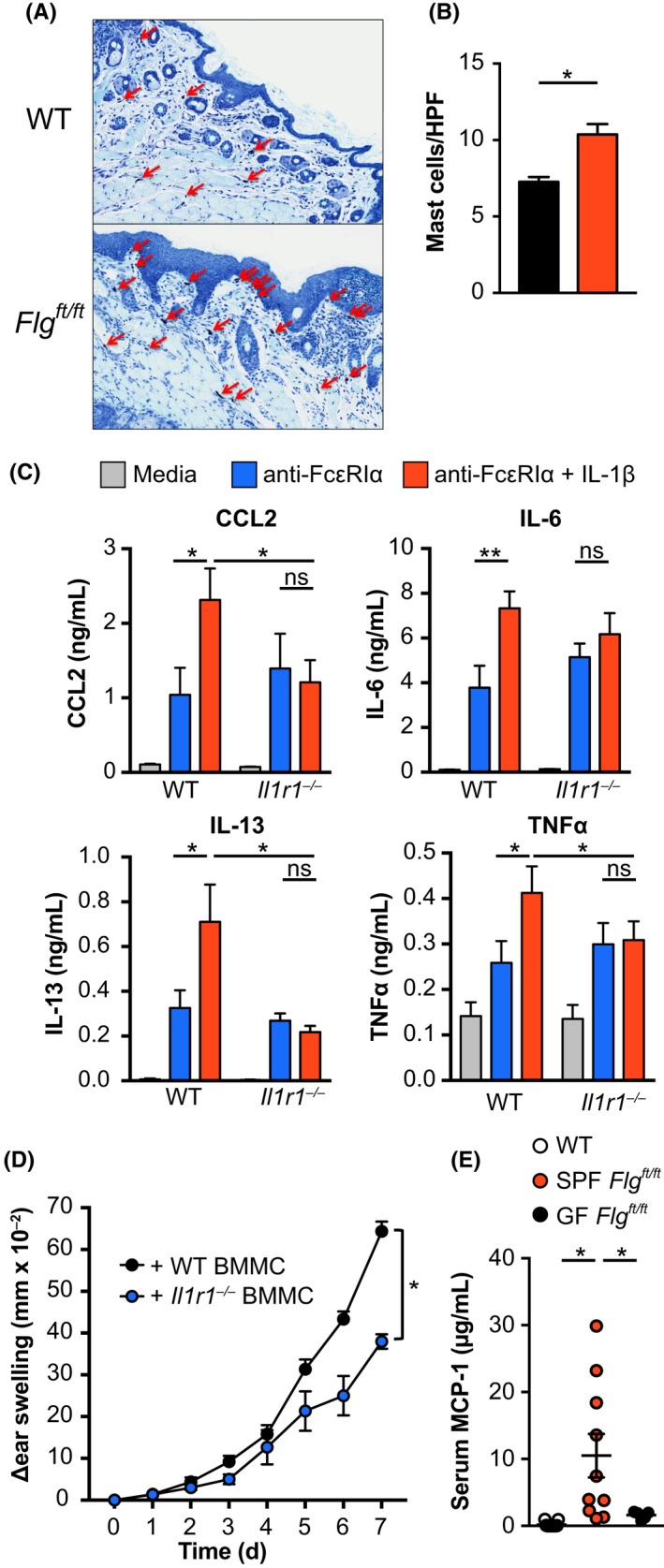
IL‐1β drives hyperactivation of mast cells in AD‐like inflammation. A, Toluidine blue staining of wild‐type (upper panel) and *Flg^ft/ft^* (lower panel) skin. Red arrows indicate connective tissue mast cells. B, Quantification of mast cells per HPF from toluidine blue stained skin samples. Bar graphs show the mean + SEM from 8 mice per group. *P < 0.05, *t*test. D, Bone marrow–derived mast cells from wild‐type (left) and *Il1r1^−/−^* (right) mice were stimulated with media (light gray) or plate‐bound anti‐FcεRIα (10 µg/mL) in the presence (red) or absence (blue) of recombinant IL‐1β (10 ng/mL). CCL2, IL‐6, IL‐13, and TNFα were measured by ELISA. Bars show the mean + SEM of nine replicates from three independent experiments. *P < 0.05. E, Wild‐type (black) and IL‐R1*^−/−^* (blue) mast cells were transferred intradermally into the ears of *Kit^W‐sh^Flg^ft/ft^* mice. Six weeks later, MC903 or 100 EtOH was applied onto the ears and ear thickness was measured daily. Graph shows the mean + SEM of 5 mice per group from two experiments. *P < 0.05, *t* test of AUC. F, Serum mast cell protease 1 levels from adult (10 wk) wild‐type (open circles), *Flg^ft/ft^* (red), and germ‐free *Flg^ft/ft^* (black) mice. *P < 0.05

A study in mice with NLRP3‐dependent skin inflammation analyzed the interplay between microbiota, TNFα, and IL‐1β and demonstrated a critical role for mast cells in skin inflammation.^66^ Histological analysis of the skin of adult *Flg^ft/ft^* mice showed elevated numbers of connective tissue mast cells (Figure 6A,B). As we observed significant amelioration in response to IL‐1β deficiency, we were interested whether IL‐1β was directly promoting mast cell activation and inflammation in *Flg^ft/ft^* mice. Therefore, we generated bone marrow–derived mast cells from wild‐type and *Il1r1^−/−^* donors and stimulated them through FcεRI in the presence and absence of IL‐1β costimulation (Figure 6C). While FcεRI‐crosslinking induced the release of pro‐inflammatory IL‐6 and TNFα, as well as CCL2 and IL‐13, costimulation with IL‐1β further increased cytokine release in an IL‐1R1‐dependent manner (Figure 6C). This result suggests a hyperresponsive phenotype of mast cells and a pro‐inflammatory role in dermatitis exacerbation in response to IL‐1β.

In order to confirm the pathogenic role of IL‐1β‐responsive mast cells in skin inflammation, we transferred wild type‐ or *Il1r1^−/−^*‐derived BMMC into the ears of mast cell–deficient *Kit^W‐sh^* mice^67^ on a *Flg^ft/ft^* background (Figure 6D). Indeed, when we induced inflammation in these mice using MC903, IL‐1β‐responsive mast cells were sufficient to aggravate inflammation (Figure 6D). Interestingly, germ‐free *Flg^ft/ft^* mice had significantly decreased serum levels of IgE compared to their SPF counterparts (Figure 2H) and—lacking the IgE‐mediated activation of mast cells—also showed decreased levels of MCP‐1 (Figure 6F). Importantly, GF mice showed reduced expression of IL‐1β further reducing activation of mast cells (Figure 2I). These data indicate that IL‐1β‐mediated hyperactivation of mast cells contributes significantly to dermatitis in *Flg^ft/ft^* mice.

## 4 DISCUSSION

In the present study, we have shown that the development of skin inflammation in *Flg^ft/ft^* mice is independent of group 2 innate lymphoid cells but requires an interplay between the microbiome, IL‐1β, and mast cells. ILC2 are implicated in AD, with AD skin lesions showing higher expression of ILC2 genes *RORA, IL1R1, IL17RB, TSLPR*, and *AREG*, and increased numbers of ILC2.^25^, ^27^ We have reported elevated ILC2 in skin blisters of AD patients with mutation in *FLG*.^11^ Here, mice deficient in ILC2 developed significantly ameliorated acute skin inflammation but despite the higher numbers of ILC2 present in the skin of *Flg^ft/ft^* mice,^11^ ILC2 play a redundant role in the pathogenesis of chronic dermatitis. While MC903‐induced ILC2 function could be targeted by anti‐IL‐18,^68^ we show that chronic inflammation is independent of IL‐18, further corroborating the observed redundancy of ILC2.^69^, ^70^ Although ILC2 may contribute to genesis of acute skin inflammation, their increase in numbers under chronic inflammatory conditions is probably a consequence of an overall increase in infiltrating immune cell populations.

A similar general increase of type 2‐ and 17‐associated cytokine production is observed in the inflamed skin of *Flg^ft/ft^* mice; however, no functional roles for individual cytokines were observed in mutant mice. This is perhaps surprising as IL‐4 and IL‐13 are known to be involved upstream of filaggrin in the development of AD by promoting skin barrier disintegration^71^, ^72^ and dermal fibrosis^73^—possibly via TSLP.^74^ IL‐5 and IL‐9 are effector cytokines promoting single aspects of AD, such as eosinophilia,^56^ neuronal stimulation,^75^ and survival of T cells and ILC2.^76^ IL‐17A can promote skin inflammation^77^ and was shown to decrease expression of filaggrin and tight junction proteins.^78^ Similarly, IL‐22 is able to regulate keratinocyte function.^63^, ^79^ Despite these cytokines being upregulated in the skin, the single knockout of each cytokine did not alleviate disease. This highlights that single target therapy may not be useful to therapeutically deplete cytokines during chronic disease. In this context, dupilumab, targeting the IL‐4Rα‐chain and thereby the actions of IL‐4 and IL‐13, has recently been approved by the FDA for the treatment of moderate‐to‐severe AD patients (reviewed in^80^) and is proving to be efficacious.^81^, ^82^ Interestingly, in the context of this study, in patients undergoing dupilumab treatment IL‐1β was shown to be considerably downregulated,^83^ while markers of skin integrity, including *FLG,* are restored.^84^

We show that chronic dermatitis in *Flg^ft/ft^* mice developed independently of IL‐1α but was instead dependent on IL‐1β. Importantly, treatment with anti‐IL‐1β ‐antibody could alleviate MC903‐induced skin flares supporting findings that canakinumab can prevent TSLP release from keratinocytes.^85^ In a recent study, it was shown that 6‐8‐week‐old *Flg^ft/ft^* mice without overt dermatitis constitutively expressed increased amounts of IL‐1α in the epidermis but 21 days after acute mechanical skin injury released less IL‐1α and more IL‐1β.^86^ These results indicate important, but divergent, roles for IL‐1 family cytokines in acute and chronic dermatitis. One of our next steps will therefore include the analysis what impact IL‐1‐family members have on expression of key skin barrier proteins.

In *Flg^ft/ft^* mice, we observed that IL‐1β enhances FcεRI‐mediated signaling in mast cells confirming previous reports.^87^ Recently, it was shown that mast cells with a mutated NLRP3 inflammasome and higher caspase‐1 activity produced IL‐1β in response to TNFα or LPS stimulation.^66^ Using *kit^w‐sh^Flg^ft/ft^* mice as mast cell–recipient mice, we show that IL‐1β‐responsive mast cells promote inflammation via IL‐1R1‐signaling. Because *kit^w‐sh^* mice certainly have phenotypes independent of mast cell deficiency,^88^ further studies using more sophisticated models of mast cell deficiency are required to analyze their in vivo function in mice with skin barrier defects.

Treatment with the NLRP3‐inflammasome inhibitor MCC950 did not ameliorate inflammation in our multifactorial model of AD when we triggered an acute inflammatory response with MC903. Furthermore, inflammation was unaltered in NLRP3‐deficient, ASC‐deficient, and AIM2‐deficient *Flg^ft/ft^* mice. Therefore, IL‐1β processing occurs independent of the NLRP1, NLRP3, NLRP6, and AIM2 inflammasome. To date, we cannot rule out a role for the NLRC4 inflammasome (or NAIP‐NLRC4) that can directly recruit caspase‐1. In a recent study, NLRC4 was found in the scale extracts of psoriasis patients; however, no inflammasome components (including NLRC4) were found in atopic dermatitis patients.^89^ Inflammasome‐independent processing of IL‐1β has been observed in a variety of settings (reviewed in^90^). In particular, neutrophil‐ and mast cell–derived proteases play prominent roles in the extracellular processing of IL‐1‐family cytokines,^91^, ^92^ with human mast cell protease 1 shown to process pro‐IL‐1.^93^ Increased mast cell chymase activity may therefore contribute to IL‐1β maturation in the *Flg^ft/ft^* model; however, further investigations are required to fully understand the role of mast cell proteases in AD.

Skin microbiome sequencing revealed a shift toward pathogenic staphylococci, which has been reported in AD patients.^30^ While the microbiota in flaky tail mice induced IL‐17A‐mediated neutrophilia, we could show that in *Flg^ft/ft^* mice IL‐17A was redundant.^33^ Indeed, IL‐17A‐mediated inflammation is observed in *Tmem79^ft/ft^* (Saunders et al, unpublished) but not in *Flg^ft/ft^* mice. Our results indicate that the pathogenic microbiome present in neonates imprinted a hyperresponsive phenotype in mast cells. This phenotype is maintained when adult mice were treated with antibiotics. When we treated neonate *Flg^ft/ft^* mice with antibiotics before inflammation is established, or when mice are raised in GF conditions, the subsequent adult dermatitis phenotype is significantly ameliorated. A recent study in a cohort of newborn children suggests that early colonization with commensal staphylococci genera protects from AD and that microbiotic changes only occur after the onset of AD.^94^ Therefore, the *Flg^ft/ft^* mouse model provides a basis for future investigations into the extrinsic factors and intrinsic mechanisms of neonatal inflammation before AD‐like inflammation develops. While the broad application of state‐of‐the‐art techniques gives a comprehensive analysis of the AD‐like inflammation in mutant mice on the *Flg^ft/ft^* background, future approaches need to target the separate pathways leading to disease and untangle the relative contribution to pathogenesis.

In summary, we revealed a critical role of IL‐1β in the initiation and maintenance of skin inflammation in a mutant mouse model of defective skin barrier. Further investigations will be required to assess the contribution of IL‐1β‐responsive mast cells, as well as roles of the integrity of the skin barrier and interplay of the microbiome, in the generation of inflammation in AD patients. These endeavors will lead to the development of novel management options for children suffering from AD.

## CONFLICTS OF INTEREST

The authors declare that they have no conflicts of interest.
