# Supplementary material for: Spontaneous atopic dermatitis in mice with a defective skin barrier is independent of ILC2 and mediated by IL‐1β
Source: Allergy. 2019 Apr 29;74(10):1920–33. doi: 10.1111/all.13801 (PMC6850072; doi:10.1111/all.13801)
Supplement: Supplementary file 1 [file ALL-74-1920-s001.pdf]

Material and Methods

Flow cytometry

Cells were stained with Live/Dead aqua (Life Technologies) in PBS for 30 min at 4˚C and Fc receptors were then blocked with 10 μg/ml purified anti-CD16/32 antibody (clone 93; eBioscience) in FACS buffer (PBS, 2% FBS, and Na3N) for 15 min at 4˚C. Cells were incubated in FACS buffer for 30 min at 4˚C with combinations of antibodies listed in Supplementary Table 1.

ILC2 were defined as Lin−CD45+CD90+ST2+KLRG1+, mast cells as CD45+CD117+IgE+/FcεRIα+, eosinophils as CD45+Siglec-F+CD11b+, basophils as CD45+IgE+CD200R3+CD117. Cells were acquired on a DAKO CyanADP machine and analyzed with FlowJo software (Treestar).

MC903-induced skin inflammation

MC903 (Calcipotriol) was dissolved in 100% ethanol and 4 nmol in 20 μl applied daily onto the ears of wildtype mice for a total of 5-7 days, as previously described (Li, 2006). *Flg*$^{ft/ft}$ mice received low-dose (1 nmol) MC903 to account for increased skin permeability. Ear thickness was measured daily with a thickness gage (Mitutoyo).

Antibody treatment of Flg$^{ft/ft}$ *mice*

Flgft/ft mice received daily intraperitoneal injections of 200 μg of anti-IL-1α (clone ALF-161; BioXCell, USA), anti-IL-1β (clone B122; BioXCell, USA) and isotype control mAb (clone MOPC-21; BioXCell, USA) on Day -1 to Day 4. Analysis of mice was performed 24 hours after the last MC903 application.

Histology

Skin tissue samples were fixed in 10% neutral buffered formalin, embedded in paraffin, and 4-μm slices were cut on a microtome before staining with hematoxylin/eosin, chloroacetate esterase, or toluidine blue. Pictures were acquired on an Aperio ScanScope at 20x original magnification.

Transepidermal water loss

Measurement of transepidermal water loss (TEWL) was measured using a Courage and Khazaka Tewameter TM210 (EnviroDerm, Evesham, UK), as previously described (Fallon, 2009). TEWL was recorded at room temperatures of 19-21˚C and 50 +/-5 % relative humidity.

Human suction blister studies

Studies on AD patients were as described (Salimi, 2013; Saunders, 2016). Suction blistering was performed on patients with informed written consent, and sample use was given ethical approval from the NRES Committee South Central, United Kingdom. IL-1β in Blister fluid was analyzed with the MAGPIX Multiplex Array (Luminex, Austin, Tex), according to the manufacturer's instructions.

Measurement of proteins

Commercial ELISA kits were used for quantification of total serum IgE (BD Pharmingen), Mcpt1, and cytokines (R&D Systems, Minneapolis, MN). Skin tissue was homogenized in a buffer containing 1 X PBS, 2% fetal bovine serum and 0.5% cetyltrimethylammonium bromide, using an IKA T10 Basic Ultra-Turrax homogenizer. Homogenates were centrifuged at 13000 g for 15 min. Cytokines were determined using ELISA kits or V-PLEX Mouse Cytokine kit (Meso Scale Discovery). Cytokine levels were normalized to total protein following BCA assay.

Antibiotics treatment

Flg$^{ft/ft}$ mice were treated with a broad-spectrum antibiotic (ABX) treatment protocol (Hill, 2010; Floudas, 2017). Pregnant *Flg*$^{ft/ft}$ mice were administered ABX orally daily from for 10 consecutive days, from E14 to P5, with vancomycin (0.5mg/ml), neomycin (1mg/ml), metronidazole (1mg/ml), ampicillin (1mg/ml) and gentamicin (1mg/ml) (Sigma-Aldrich) in autoclaved water. Cages were maintained on ABX-supplemented drinking water - metronidazole (0.5 mg/ml) and ciprofloxacin (0.125 mg/ml) – until weaning at P21. For ABX treatment of adult mice, 8-9 week old *Flg*$^{ft/ft}$ mice were scored for skin inflammation and randomly assigned to ABX or control groups. Mice were treated with ABX regime and *control mice* were treated orally with autoclaved water daily by oral gavage for 10 days.

Supplemental references

Fallon PG, Sasaki T, Sandilands A, et al. A homozygous frameshift mutation in the mouse Flg gene facilitates enhanced percutaneous allergen priming. *Nat Genet.* 2009;41(5):602-608.

Floudas A, Saunders SP, Moran T, et al. IL-17 Receptor A Maintains and Protects the Skin Barrier To Prevent Allergic Skin Inflammation. *J Immunol.* 2017;199(2):707-717.

Hill DA, Hoffmann C, Abt MC, et al. Metagenomic analyses reveal antibiotic-induced temporal and spatial changes in intestinal microbiota with associated alterations in immune cell homeostasis. *Mucosal Immunol.* 2010;3(2):148-158.

Li M, Hener P, Zhang Z, Kato S, Metzger D, Chambon P. Topical vitamin D3 and low-calcemic analogs induce thymic stromal lymphopoietin in mouse keratinocytes and trigger an atopic dermatitis. *Proc Natl Acad Sci U S A.* 2006;103(31):11736-11741

Salimi M, Barlow JL, Saunders SP, et al. A role for IL-25 and IL-33-driven type-2 innate lymphoid cells in atopic dermatitis. *J Exp Med.* 2013;210(13):2939-2950.

Saunders SP, Moran T, Floudas A, et al. Spontaneous atopic dermatitis is mediated by innate immunity, with the secondary lung inflammation of the atopic march requiring adaptive immunity. *J Allergy Clin Immunol.* 2016;137(2):482-491.

Supplemental table 1:
Antibodies used for flow cytometry staining

	Specificity	Clone	Fluorochrome	Company
lineage	CD3	17A2	eFlour 450	ebioscience
lineage	TCRb	H57-597	eFlour 450	ebioscience
lineage	CD5	53-7.3	eFlour 450	ebioscience
lineage	CD19	ebio1D3	eFlour 450	ebioscience
lineage	gd TCR	ebioGL3	eFlour 450	ebioscience
lineage	CD11b	M1/70	eFlour 450	ebioscience
lineage	CD11c	N418	eFlour 450	ebioscience
lineage	FcERIa	MAR-1	eFlour 450	ebioscience
lineage	Ter-119	TER119	eFlour 450	ebioscience
lineage	Gr-1	RB6-8C5	eFlour 450	ebioscience
lineage	F4/80	BM8	eFlour 450	ebioscience
	T1/ST2	DJ8	FITC	MD Biosciences
	CD274	MIH5	PE	ebioscience
	KLRG1	2F1	PE-eFlour 610	ebioscience
	CD25	PC61.5	APC	ebioscience
	CD45	30-F11	PerCP-Cy5.5	BD
	CD45	104	PE-CF594	BD
	Siglec-F	E50-2440	PE	BD
	MHC2	M5/114.15.2	FITC	ebioscience
	MHC2	M5/114.15.2	eFlour 450	ebioscience
	MHC2	M5/114.15.2	APC-eFlour 780	ebioscience
	CD3	145-2C11	APC	ebioscience
	CD3	145-2C11	PE-eFlour 610	ebioscience
	CD3	17A2	FITC	ebioscience
	CD4	RM4-5	APC	ebioscience
	CD4	RM4-5	APC-eFlour 780	ebioscience
	CD4	RM4-5	PerCP-Cy5.5	ebioscience
	CD11b	M1/70	PerCP-Cy5.5	ebioscience
	CD11c	N418	PE-Cy7	ebioscience
	CD11c	N418	eFlour 450	ebioscience
	F4/80	BM8	FITC	ebioscience
	F4/80	BM8	APC	ebioscience
	CD200R3	Ba13	PE	BioLegend
	IgE	23G3	FITC	ebioscience
	IgE	23G3	PE	ebioscience
	CD117	2B8	PE	ebioscience
	FcERIa	MAR-1	FITC	ebioscience
	FcERIa	MAR-1	eFlour 450	ebioscience

Staining of transcription factors

FoxP3 staining buffer kit			ebioscience
GATA3	TWAJ	PE	ebioscience
Tbet	04-46	FITC	BD
Tbet	04-46	APC	BD
RORgT	Q31-378	Brillant Violet 421	BD
FoxP3	MF23	APC	BD
FoxP3	MF23	PE-CF594	BD

Supplemental figure 1

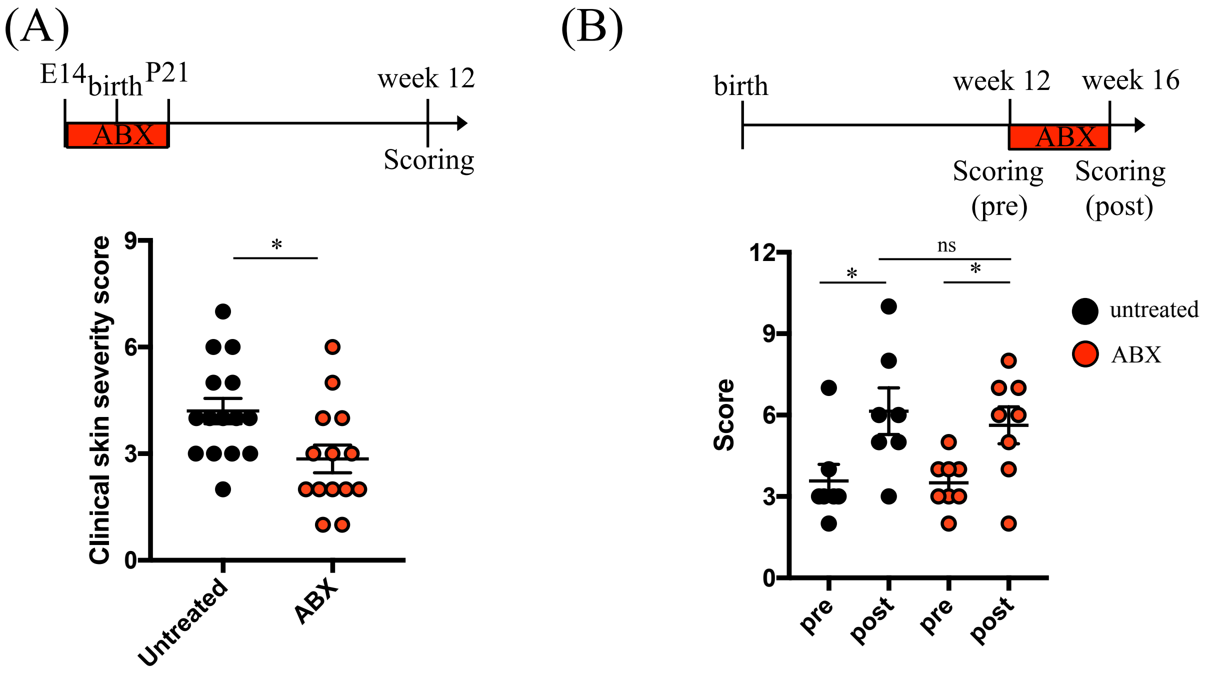

Supplemental Figure 1. Antibiotic treatment before established dermatitis ameliorates inflammation in *Flg*$^{ft/ft}$ mice. (A) Pregnant female *Flg*$^{ft/ft}$ mice and their offspring were treated with antibiotics (ABX) or not (untreated) for four weeks (E14-P21). Clinical skin severity score of the adult offspring (12 weeks later) is shown. *, P<0.05, t-test. (B) Adult (12 week old) *Flg*$^{ft/ft}$ mice were treated with antibiotics for four weeks (red) or left untreated (black). Clinical skin severity shown before (pre) and after antibiotics treatment (post) is shown. *, P<0.05, ns = not significant, t-test.

Supplemental figure 2

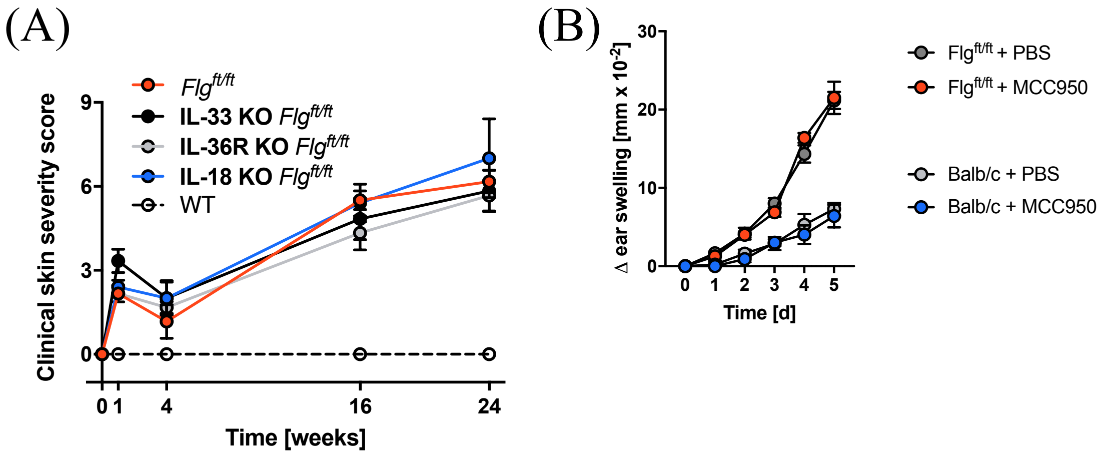

Supplemental Figure 2. Inflammasome-independent inflammation.
(A) Macroscopic clinical scoring of *Flgft/ft* (red), IL-18-/-*Flgft/ft* (black), IL-36R-/-*Flgft/ft* (light blue) and IL-33-/-*Flgft/ft* (blue) mice. Graph shows the mean+SEM of six mice per group.
(B) Balb/c and *Flg*$^{ft/ft}$ mice were treated with PBS (gray circles) or inflammasome inhibitor MCC950 (red: *Flg*$^{ft/ft}$, blue: Balb/c) and MC903 was topically applied to induce dermatitis exacerbation. Ear thickness was measured daily. Graph shows the mean+SD of 4-6 mice per group.
